# Theoretical Investigation of Geometries and Bonding of Indium Hydrides in the In_2_H_x_ and In_3_H_y_ (x = 0–4,6; y = 0–5) Series

**DOI:** 10.3390/molecules28010183

**Published:** 2022-12-26

**Authors:** Anton S. Pozdeev, Pavel Rublev, Steve Scheiner, Alexander I. Boldyrev

**Affiliations:** Department of Chemistry and Biochemistry, Utah State University, 0300 Old Main Hill, Logan, UT 84322-0300, USA

**Keywords:** indium hydrides, bonding, AdNDP analysis, indium compounds

## Abstract

Boron hydrides have been an object of intensive theoretical and experimental investigation for many decades due to their unusual and somewhat unique bonding patterns. Despite boron being a neighboring element to carbon, boron hydrides almost always form non-classical structures with multi-center bonds. However, we expect indium to form its interesting molecules with non-classical patterns, though such molecules still need to be extensively studied theoretically. In this work, we investigated indium hydrides of In_2_H_x_ (x = 0–4,6) and In_3_H_y_ (y = 0–5) series via DFT and ab initio quantum chemistry methods, performing a global minimum search, chemical bonding analysis, and studies of their thermodynamical stability. We found that the bonding pattern of indium hydrides differs from the classical structures composed of 1c-2e lone pairs and 2c-2e bonds and the bonding pattern of earlier investigated boron hydrides of the B_n_H_n+2_ series. The studied stoichiometries are characterized by multi-center bonds, aromaticity, and the tendency for indium to preserve the 1c-2e lone pair.

## 1. Introduction

Classical organic chemistry is based on a carbon atom in a valence state of IV. This element usually tends to form 2c-2e bonds with carbon and other elements. Despite boron and carbon being neighboring elements, boron tends to form multi-center bonds [1,2,3]. In particular, it was shown [4] that the B_n_H_n+2_ classical structures become less stable along the series because boron avoids expected sp^2^-hybridization. Despite the rich chemistry of boron hydrides being well-studied [5,6,7,8,9], our knowledge of indium hydride compounds is minimal. Only a small number of indium hydrides and their derivatives have been previously synthesized [10,11] or theoretically investigated [12,13,14]. To our knowledge, almost no conformational search procedures have been applied for indium hydrides with several indium atoms; therefore, the possible non-trivial properties of indium hydrides have been missed.

Being a relatively heavy element, indium atoms have less preference for any type of sp hybridization in hydrogen compounds compared to boron and aluminum. One of the main reasons for this is related to weaker hydrogen–indium orbital overlaps and, therefore, lower interaction: dissociation energies are 3.42 eV and 2.48 eV for B-H and In-H bonds, respectively [15]. Another important reason is a more significant energy gap between 5s and 5p orbitals induced by complete electronic 4d-subshell and the subsequent nucleus charge screening effect. It can be vividly seen from the difference between excitation energies ^2^P^o^ → ^4^P of boron and indium atoms [16,17].

Thus, are the indium and boron hydride structures similar or different? Are classical arrangements with only 1c-2e lone pairs and 2c-2e bonds possible for indium hydrides? In this work, we investigated the nature of In_2_H_x_ (x = 0–4,6) and In_3_H_y_ (y = 0–5) compounds using the Coalescence Kick global optimization techniques, chemical bonding AdNDP analysis, thermodynamic stability toward H_2_ dissociation to answer the abovementioned questions.

## 2. Results and Discussions

### 2.1. Global Geometry Optimization and Bonding Analysis

Initially, we performed the CK search for In_2_H_x_ and In_3_H_y_ (x = 0–4; y = 0–5). The investigated global minima structures are shown in Figure 1. The obtained geometry of the global minimum and energy ordering for low-lying isomers of In_2_H_2_ stoichiometry is in entire agreement with the previous investigation [18]. Other low-lying geometries are given in the SI file (Figures S1–S11).

According to the AdNDP analysis, completely unsaturated structures In_2_ and In_3_ have similar bonding patterns. In the case of the In_2_ molecule in the first triplet state, we found two 1c-2e lone pairs, one 2c-1e σ-bond with ON = 0.99e, and one 2c-1e π-bond with ON = 0.99e, where “ON” stands for occupancy number, and “e” reflects that occupancy number is related to the number of electrons. 

**Figure 2:**
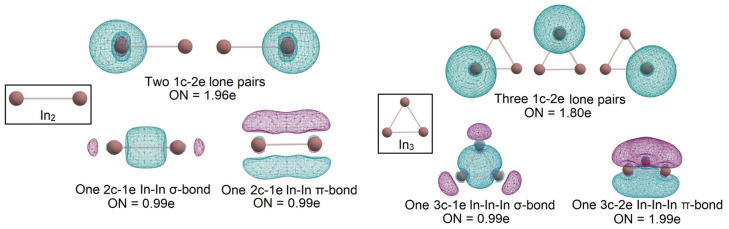
The chemical bonding pattern of In_2_ and In_3_ global minimum structures obtained by AdNDP analysis.

For In_3_, we investigated three 1c-2e lone pairs with ON = 1.80e, 3c-2e π-bond with ON = 2.00e, and 3c-1e σ-bond with ON = 0.98e for an unpaired electron. We assign a molecule as being doubly aromatic (i.e., π and σ aromatic; Figure 2). Negative NICS_ZZ_ values at different distances from XY-plane can be considered another argument for the aromaticity of In_3_ [19]. For example, in the case of benzene NICS_ZZ_(0) = −15.199 ppm, NICS_ZZ_(1) = −29.517 ppm, NICS_ZZ_(2) = −17.315 ppm, NICS_ZZ_(3) = −8.014 ppm showing the presence of π-aromaticity. In the case of In_3_ NICS_ZZ_(0) = −2.146 ppm, NICS_ZZ_(1) = −17.565 ppm, NICS_ZZ_(2) = −17.139 ppm, NICS_ZZ_(3) = −9.760 ppm. For the In_3_ cluster, the absolute values of NICS_ZZ_(0) and NICS_ZZ_(1) are much smaller than those for benzene. However, the values of NICS_ZZ_(2) and NICS_ZZ_(3) are similar for both molecules; an explanation of the difference may be based on the types of involved orbitals. Benzene is a π aromatic molecule, but In_3_ is a doubly π and σ aromatic, which indicates the significant difference of NICS_ZZ_(0) and NICS_ZZ_(1) values near the molecular plane, where σ orbitals influence more significantly. It is worth mentioning that an unpaired electron on a σ-like orbital provides a more energetically stable state than a state with an unpaired electron on a π-like orbital. The difference between those electronic states is 4.12 kcal/mol and was obtained using the Δ-CCSD(T)/cc-pVTZ(-PP) level of theory. The same procedure was performed for the In_2_ molecule. An alternative electronic state with two occupied perpendicular single-occupied 2c-1e π-bonds in the Slater determinant was investigated. This state was less stable by 5.8 kcal/mol at the Δ-CCSD(T)/cc-pVTZ(-PP) level of theory. 

**Figure 3:**
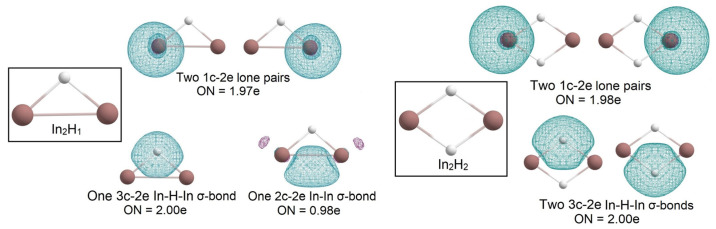
The chemical bonding pattern of In_2_H_1_ and In_2_H_2_ global minimum structures obtained by AdNDP analysis.

Further “hydrogenation” evolution of the In_2_H_x_ series reveals some features of indium hydride compounds. Besides 1c-2e lone pairs, in In_2_H_1,_ we observed 3c-2e In-H-In σ-bond with occupation number ON = 2.00e and 2c-1e In-In σ-bond with ON = 0.98e, but in In_2_H_2_ we did not find 2c-2e bonds—only two 1c-2e lone pairs and two 3c-2e In-H-In σ-bonds with ON = 2.00e (Figure 3). Thus, upon “hydrogenation,” indium saves its lone pair instead, forming the 2e-2c classical In-In σ-bond.

**Figure 4:**
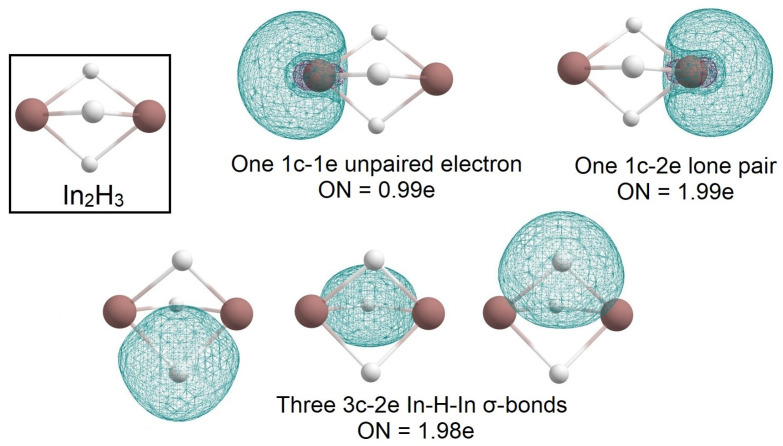
The chemical bonding pattern of the In_2_H_3_ global minimum structure obtained by AdNDP analysis.

In the case of the In_2_H_3_ molecule, the AdNDP algorithm allowed us to describe the bonding pattern as three 3c-2e In-H-In σ-bonds with ON = 1.98 e, one 1c-1e unpaired electron with ON = 0.99e on one indium atom, and a 1c-2e lone pair on another indium atom with ON = 1.99e (Figure 4).

**Figure 5:**
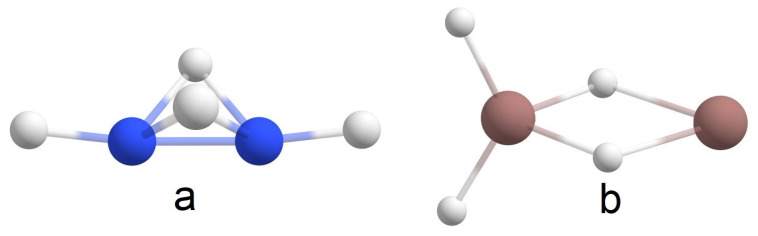
The comparison of the global minimum structures for (**a**) B_2_H_4_ (according to [4]) and (**b**) In_2_H_4_.

**Figure 6:**
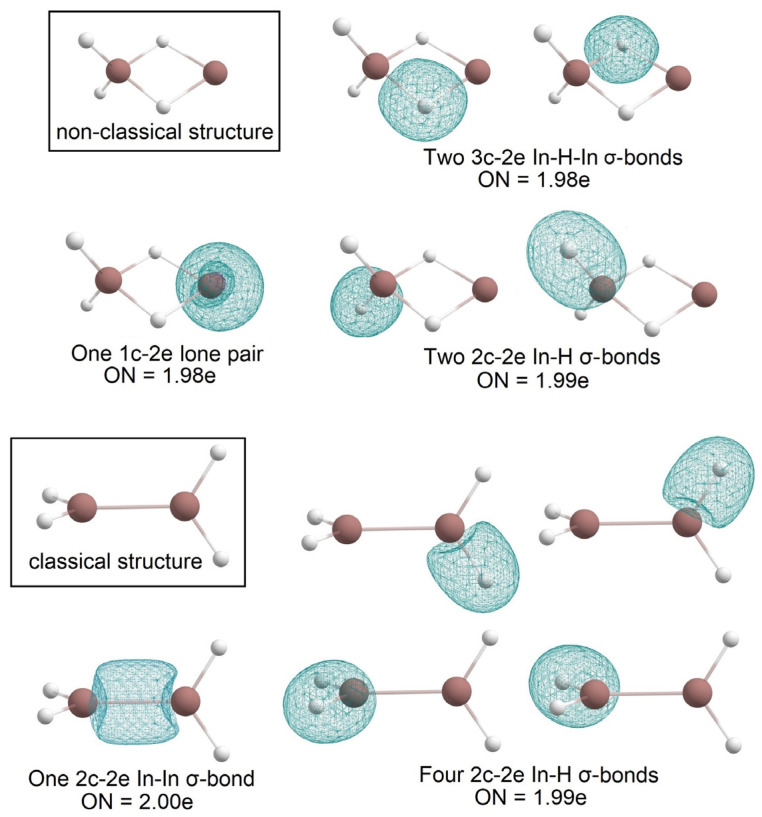
The chemical bonding pattern of In_2_H_4_―non-classical structure (global minimum) and classical structure obtained by AdNDP analysis.

According to [4], structure **a** in Figure 5 is the global minimum of B_2_H_4_. In the present study, structure **b** (Figure 5) is the global minimum for In_2_H_4_. Among others, one significant difference between these two molecules is that in the case of B_2_H_4,_ there is one B-B 2c-2e σ-bond and no 1c-2e lone pairs, but In_2_H_4_ has two 3c-2e In-H-In σ-bonds with ON = 1.98e, one 1c-2e lone pair on one indium atom, and no In-In 2c-2e σ-bonds (Figure 6). Moreover, our attempts to find a similar In_2_H_4_ structure to the structure in Figure 5a at different theoretical levels were unsuccessful. Thus, this direct comparison of the global minimum structures of B_2_H_4_ and In_2_H_4_ demonstrates the difference in indium and boron hydride bonding patterns. Both have non-classical structures (i.e., they have delocalized multi-center 2e bonds). However, all boron valence electrons participate in bonding, whereas indium tends to save its lone pair. 

An earlier theoretical study of classical and non-classical structures of B_2_H_4_ in [4] showed that the non-classical structure is more stable by 2.9 kcal/mol. In this work, the CK search allowed us to find a classical structure for In_2_H_4_. The AdNDP analysis reveals that both indium atoms are connected via 2c-2e σ-bonds with ON = 2.00e, and all other In-H bonds are 2c-2e σ-bonds with ON = 1.99e. The energy difference between the classical and non-classical structures is 10.8 kcal/mol at the QRO-CCSD(T)/cc-pVTZ(-PP)//U-TPSSh/def2-TZVPP level of theory. The energy difference is too big to suggest a competition of the classical structure with the global minimum structure. The bonding analysis of the two structures is shown in Figure 6. 

**Figure 7:**
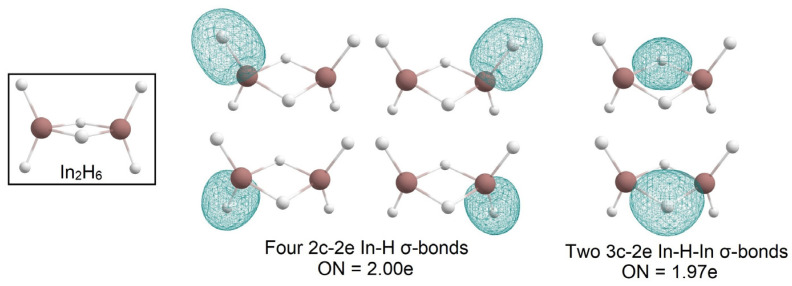
The chemical bonding pattern of the In_2_H_6_ global minimum structure obtained by AdNDP analysis.

Additionally, we decided to investigate the In_2_H_6_ species as an analog of the famous diborane molecule. The CK algorithm found only one non-dissociated structure of this stoichiometry; it totally resembles the structure of B_2_H_6_. It indicates the stability of the motif. The structure and bonding analysis are presented in Figure 7. We found four 2c-2e In-H σ-bonds with ON = 2.00e and two 3c-2e In-H-In σ-bonds with ON = 1.97e.

**Figure 8:**
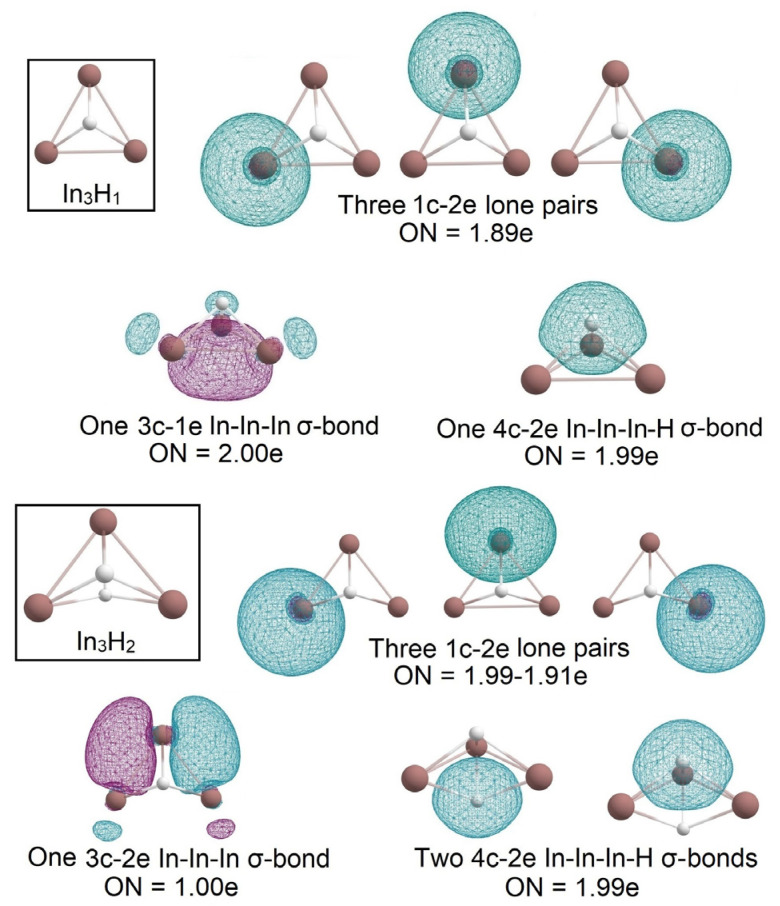
The chemical bonding pattern of In_3_H_1_ and In_3_H_2_ obtained by AdNDP analysis.

In the In_3_H_y_ series, we observed the gradual “modification” of the In_3_ triangular cluster upon “hydrogenation”. In_3_H and In_3_H_2_ have similar structures where each hydrogen is connected with the In_3_ cluster via 4c-2e In-In-In-H σ -bond with ON = 1.99e (Figure 8). In_3_H retains σ-aromaticity with an occupation number close to ideal 2.00e. Calculated values of NICS_ZZ_ for In_3_H_1_ are NICS_ZZ_(0) = +3.804 ppm, NICS_ZZ_(1) = −12.155 ppm, NICS_ZZ_(2) = −14.271 ppm, NICS_ZZ_(3) = −8.235 ppm. A positive value near the center of a molecule with a rapid decrease of NICS_zz_ value as the distance increases can be evidence of σ-aromaticity [20]. Due to hydrogen atom incorporation, the absolute values of NICSzz of In_3_H_1_ are smaller than for In_3_. For In_3_H_2_, we found an interesting bonding pattern of 3c-1e In-In-In σ-bond with ON = 1.00e; the AdNDP analysis reveals that it consists of two p_x_ orbitals and one perpendicular p_y_ orbital. 

**Figure 9:**
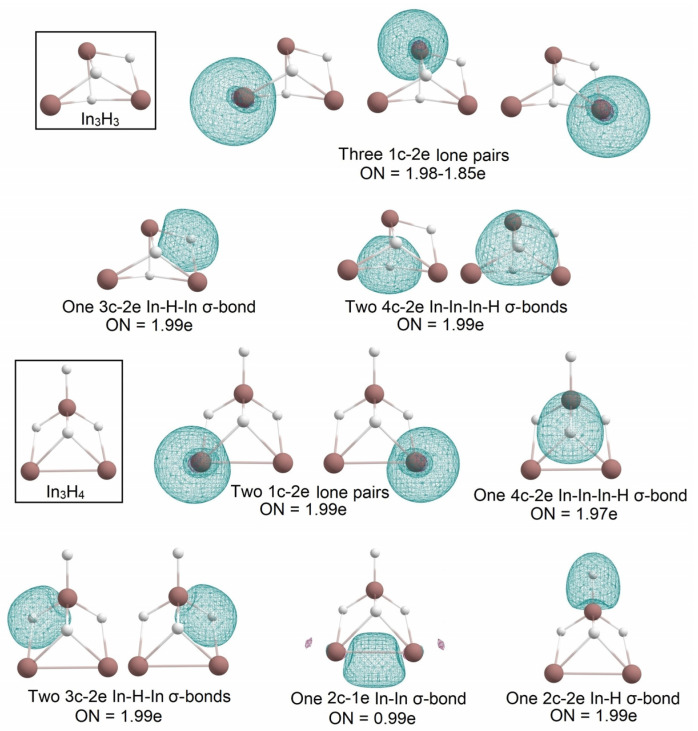
The chemical bonding pattern of In_3_H_3_ and In_3_H_4_ obtained by AdNDP analysis.

The 4c-2e In-In-In-H σ-bond appeared to be stable for the In_3_H_y_ series. In the In_3_H_3_ cluster, the bonding motif of the In_3_H_2_ structure was saved, and the third H atom was introduced in the cluster as a bridged atom in a 3c-2e In-H-In σ-bond with ON = 1.99e. This bonding pattern allowed In_3_H_3_ to keep three 1c-2e lone pairs on each indium atom with ON = 1.98–1.85e. Further “hydrogenation” leads to In_3_H_4_ losing one 4c-2e In-In-In-H σ-bond and one 1c-2e lone pair. It has two 1c-2e lone pairs, one 2c-1e In-In σ-bond with ON = 0.99e, one 2c-1e In-H σ-bond with ON = 0.99e, two 3c-2e In-H-In σ-bonds with ON = 1.99e, and one 4c-2e In-In-In-H σ-bond with ON = 1.97e (Figure 9).

**Figure 10:**
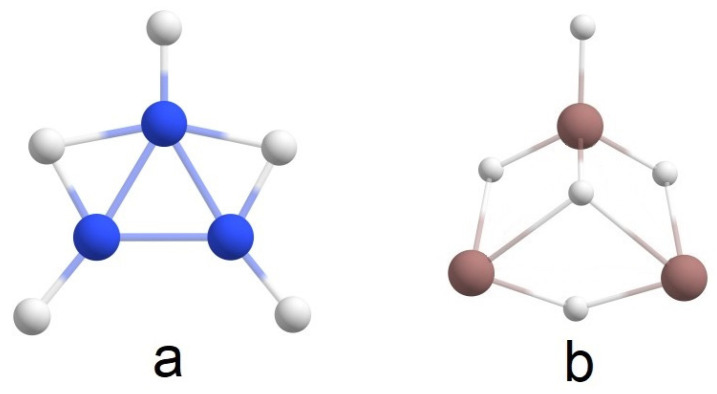
The comparison of the global minimum structures for (**a**) B_3_H_5_ (according to [4]) and (**b**) In_3_H_5_.

**Figure 11:**
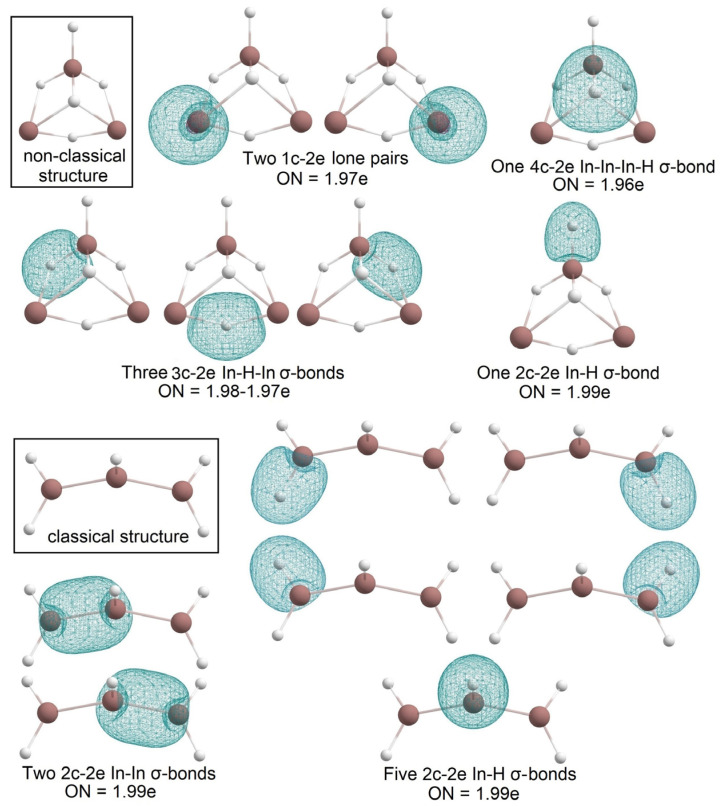
The chemical bonding pattern of In_3_H_5_―non-classical structure (global minimum) and classical structure.

As in the case of In_2_H_4_ and B_2_H_4_, the global minimum structures of In_3_H_5_ and B_3_H_5_ [4] are different. Both systems are presented in Figure 10. B_3_H_5_ has three 2c-2e B-H terminal σ-bonds, two bridged B-H-B σ-bonds, and three B atoms that are bonded via 3c-2e B-B-B σ-bond and a 3c-2e B-B-B π-bond, so there are no 1c-2e lone pairs again. In In_3_H_5_, two indium atoms save 1c-2e lone pairs; there is one In-H 2c-2e terminal σ- bond with ON = 1.99e, three bridged 3c-2e In-H-In σ-bonds with ON = 1.98–1.97e, and one 4c-2e In-In-In-H σ-bond with ON = 1.96e (Figure 11). Thus, for boron clusters, aromaticity is found for both saturated and non-saturated structures [21], but for indium, we observed aromaticity only for the most unsaturated hydrides. It is another demonstration of the difference between the patterns of boron and indium hydrides. 

The low-lying classical In_3_H_5_ structure (Figure 11) is about 17.2 kcal/mol higher than the global minimum structure; this corresponds to the trend previously observed for B_n_H_n+2_ series [4] (i.e., classical structures become progressively less stable along the series). The bonding pattern of the classical structure of In_3_H_5_ is similar to what was observed for the classical structure of In_2_H_4_; it has two types of bonds—2c-2e In-H σ-bond with ON = 1.99e and 2c-2e In-In σ bond with ON = 1.99e. 

### 2.2. Thermodynamic Stability Observation

To estimate the thermodynamics stability of the indium hydrides toward H_2_ dissociation, we calculated the potential energy difference of locally reoptimized structures at QRO-CCSD(T)/cc-pVTZ(-PP)//U-TPSSh/def2-TZVPP level of theory. The results of these calculations are shown in Table 1.

According to the data, almost all indium hydrides are stable toward H_2_ dissociation, except In_2_H_3_, In_2_H_6_, and In_3_H_4_. However, even in the case of these molecules, the ΔE of the dissociation reaction is very small.

## 3. Theoretical Methods 

The global minimum search was carried out using the Coalescence Kick (CK) algorithm written by Averkiev [22,23] to find a global minimum and corresponding low-lying isomers for In_2_H_x_ and In_3_H_y_ (x = 0–4,6; y = 0–5). The basic workflow of the CK algorithm consists of (1) the random placing of atoms in a sizeable Cartesian box, (2) the shift of atoms toward the center of mass until they coalesce up to the pairwise sums of pre-defined covalent radii of atoms, (3) the standard local geometry optimization using Ab Initio, DFT, or semi-empirical approach. The CK procedure is repeated several thousand times to obtain an adequate statistic, and the most stable structure is implied to be a global minimum. The CK original code is available online at the open repository [24]. 

All calculations utilizing the CK procedure were carried out using unrestricted Kohn–Sham formalism with density functional MN-15. It was chosen for its appropriate applicability to various electronic structure problems, including multireference behavior [25]. The LANL2DZ basis set was chosen for its balance of speed and accuracy [26]; additionally, the Hay–Wadt pseudopotential was applied to indium atoms to account for scalar relativity correction [27]. The specific generation size of random structures for the CK method for each stoichiometry is shown in the SI file (Table S1). Gaussian 16 was used as the main driver for the local optimization step [28] in the CK algorithm.

After the CK global minimum search, all obtained low-lying isomers in the 15 kcal/mol energy window from the global minimum were reoptimized with a more accurate def2-TZVPP basis set [29] and TPSSh density functional [30], which tends to provide better energy ordering of isomers but cannot be generously applied to any electronic problem. Obtained local minima were verified by nuclear Hessian calculation with the same method and basis set. The re-optimization step and all subsequent single-point calculations utilized the ORCA 5.03 suite [31,32].

Using obtained local minima geometries, single point energies were recalculated at the CCSD(T) level of theory with the cc-pVTZ [33] basis set on hydrogen atoms and the cc-pVTZ-PP basis set on indium atoms complemented with the SK-MCDHF-RSC pseudopotential [34,35]. The CCSD(T) approach is a gold standard for the accurate prediction of energy ordering and provides meaningful results only in conjunction with large basis sets. Quasi-restricted orbitals formalism (QRO-CCSD(T)) was employed to eliminate the influence of spin-contamination in the open-shell coupled clusters calculations. In addition, all CCSD(T) reference UHF wavefunctions were tested using UHF/UHF stability analysis based on the CIS method [36].

Thus, the final energy ordering of low-lying isomers was estimated using QRO-CCSD(T)/cc-pVTZ energies and U-TPSSh/def2-TZVPP geometries and corresponding zero-point energy corrections (ZPE). This calculation scheme will be denoted as “QRO-CCSD(T)/cc-pVTZ(-PP)//U-TPSSh/def2-TZVPP”.

It should be outlined that, due to the adiabatic treatment of the potential energy surface, the CK algorithm can be applied only for one spin state at a time. For stoichiometry with an even number of electrons, we chose a singlet multiplicity in the algorithm; for an odd number of electrons, we decided to use a doublet multiplicity. To verify that other spin states are not global minima, they were locally reoptimized in the corresponding triplet or quartet multiplicities at the U-TPSSh/def2-TZVPP level of theory. Further single point energy calculations at the QRO-CCSD(T)/cc-pVTZ(-PP) theoretical level demonstrated that in the case of all stoichiometries, except In_2_ and In_3_H_1_, the energy difference between a global minimum structure in the lowest spin multiplicity and a corresponding low-lying structure in triplet (or quartet) multiplicity was at least 15–30 kcal/mol. That allowed us to justify the choice of multiplicity in the CK algorithm. 

In the case of In_2,_ we found the triplet state to be more stable than the singlet state by 6.8 kcal/mol, which is in accordance with previous findings [37]. For In_3_H_1_, the singlet state is more stable than the locally reoptimized triplet state; however, the energy difference between them is relatively small (~2.5 kcal/mol). Therefore, we carried out an additional CK search to find the actual global minimum of the triplet state. In this way, we found the singlet state still to be more stable than the triplet state by 1.4 kcal/mol at the QRO-CCSD(T)/cc-pVTZ(-PP) level of theory. The energy differences between spin states are provided in the SI file (Table S2).

Chemical bonding for all global minimum isomers for each stoichiometry was analyzed using the adaptive natural density partitioning (AdNDP) algorithm implemented in AdNDP 2.0 [38,39] as an effective method of deciphering bonding in molecular clusters with untrivial electron delocalization. This approach is based on the Lewis idea that an electron pair is the main bonding element. The algorithm leads to the partitioning of the electron density into elements with the lowest symmetry-appropriate number of atomic centers per electron pair, which allows for the representation of an electronic structure as n-center two-electron bonds (nc-2e, n is an interval from one to the total number of atoms participating in the bond). The same procedure may be applied to open-shell systems and may recover nc-1e bonding elements. Despite the usage of the “electron pair idea,” AdNDP and its ideological predecessor NBO analyses based on the density matrix representation of the wavefunction; therefore, near doubly occupied bonding elements are obtained through chains of similarity or unitary transformations of canonical KS “wavefunction,” which is represented by single Slater determinant. Thus, the limit of two-electron occupancy per bonding element is not arbitrary and is dictated by Pauli’s exclusion principle for fermions and the primary modern approach to compose many-body wavefunctions. Thus, the AdNDP algorithm recovers classical Lewis bonding elements (i.e., 1c-2e lone-pairs and 2c-2e bonds) and the delocalized bonding elements similar to occupied canonical molecular orbitals, which are the most appropriate for this study. In this work, the density matrices for the AdNDP were obtained at the U-TPSSh/def2-TZVPP level of theory. Visualization of molecular structures and AdNDP orbitals was performed with ChemCraft [40].

In addition, the AdNDP algorithm may show molecules’ potential aromatic or anti-aromatic character. To verify it, the components of nucleus-independent chemical shift (NICSzz) corresponding to the principal z-axis perpendicular to a ring plane may be used as a good characteristic of aromaticity [40]. We calculated NICSzz(R) values (R means the distance from the center of a ring in Å units) at the TPSSh/def2-TZVPP level of theory.

## 4. Conclusions

In this work, we used the CK algorithm for the global minimum search for In_2_H_x_ and In_3_H_y_ (x = 0–4,6; y = 0–5) stoichiometry. Found global minimum geometries were used to investigate the structural evolution of In_2_ and In_3_ clusters under “hydrogenation”. We also investigated their chemical bonding pattern using the AdNDP algorithm and tested their thermodynamic stability toward H_2_ dissociation. Our analysis revealed that both boron and indium hydrates have non-classical structures with multi-center 2e bonds, and they are much more stable than classical structures with sp^2^ hybridization. Indium hydrides are characterized by non-intuitive global minimum geometries, where indium tends to save its lone pair even if it leads to the loss of a classical 2c-2e bond. Some unsaturated indium clusters have aromatic properties. We want to emphasize that the revealed difference between boron and indium hydrides is closely related to the difference in the fundamental properties of boron and indium atoms: higher excitation energy of indium atoms and less energetically favorable indium–hydrogen bond formation. 

We hope this work will inspire further theoretical and experimental investigation of these exotic indium species. 

## Figures and Tables

**Figure 1:**
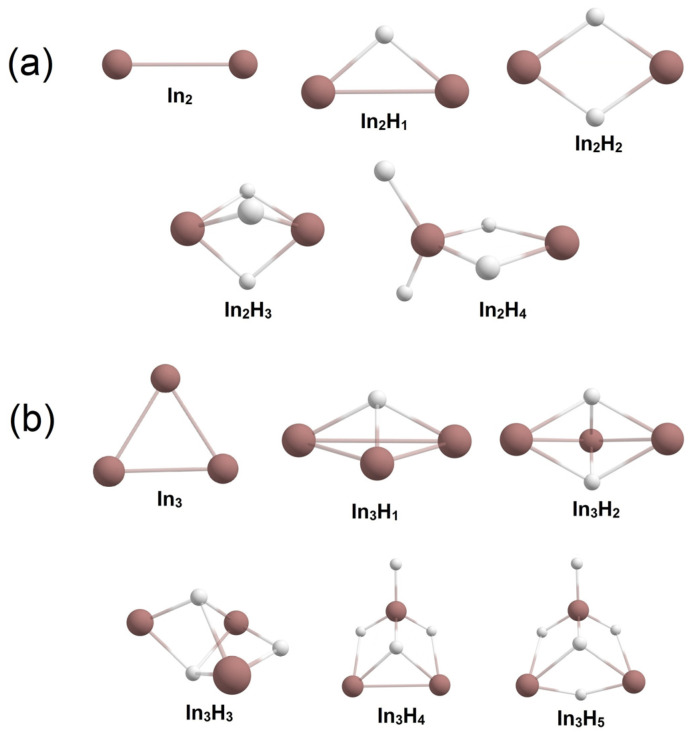
Global minimum structures of (**a**) In_2_H_x_ (x = 0–4), (**b**) In_3_H_y_ (y = 0–5).

**Table 1:** Analysis of thermodynamic stability toward H_2_ dissociation at the QRO-CCSD(T)/cc-pVTZ(-PP)//U-TPSSh/def2-TZVPP level of theory.

Stoichiometry	Decomposition Product	Stability	E^products^ − E^reagents^, kcal/mol
In_2_H_2_	In_2_ + H_2_	stable	25.2
In_2_H_3_	In_2_H + H_2_	**unstable**	**−0.1**
In_2_H_4_	In_2_H_2_ + H_2_In_2_ + 2H_2_	stable stable	4.8 30.0
In_2_H_6_	In_2_H_4_ + H_2_In_2_H_2_ + 2H_2_In_2_ + 3H_2_	**unstable** stable stable	**−2.1** 2.7 27.9
In_3_H_2_	In_3_ + H_2_	stable	12.1
In_3_H_3_	In_3_H + H_2_	stable	12.2
In_3_H_4_	In_3_H_2_ + H_2_In_3_ + 2H_2_	**unstable** stable	**−0.5** 11.6

## Data Availability

The data that support the findings of this study are available from the corresponding authors upon reasonable request.

## Supplementary Materials

The following supporting information can be downloaded at: https://www.mdpi.com/article/10.3390/molecules28010183/s1, Table S1. Population size used for CK algorithm for In_2_H_x_ and In_3_H_y_ (x = 0–4,6; y = 0–5) stoichiometries; Table S2. Spin-state energy difference; Table S3. In_2_H_x_ and In_3_H_y_ (x = 0–4,6; y = 0–5) global minimum geometries for singlet and doublet states found the lowest lying isomers for triplet and quartet states (XYZ coordinates); Table S4. NICSzz values; Figures S1–S11. Global minimum geometries and low-lying geometries with corresponding relative energies for each In_2_H_x_ and In_3_H_y_ (x = 0–4,6; y = 0–5) stoichiometry, chemical bonding patterns for global minimum geometries.

## Sample Availability

Samples of the compounds are not available from the authors.
