# Supplementary material for: Theoretical Investigation of Geometries and Bonding of Indium Hydrides in the In2Hx and In3Hy (x = 0–4,6; y = 0–5) Series
Source: Molecules. 2022 Dec 26;28(1):183. doi: 10.3390/molecules28010183 (PMC9822201; doi:10.3390/molecules28010183)
Supplement: Supplementary file 1 [file molecules-28-00183-s001.zip › molecules-2085461-supplementary.pdf]

Electronic Supplementary Information

Theoretical Investigation of Geometries and Bonding of Indium Hydrides in the In_2H_x and In_3H_y (x = 0–4,6; y = 0–5) Series

Anton S. Pozdeev[1], Pavel Rublev, Steve Scheiner and Alexander I. Boldyrev *

Department of Chemistry and Biochemistry, Utah State University, 0300 Old Main Hill, Logan, UT 84322-0300, USA
* Correspondence: a.i.boldyrev@usu.edu

Contents

Table S1. Population size used for CK algorithm 2
Table S2. Spin-states' energy difference 2
Table S3. Global minimum geometries in XYZ coordinates 2
Table S4. Calculated $NICS_{zz}$ values 6
AdNDP chemical bonding patterns and energy comparison of low-lying isomers..7

Table S1. Population size used for CK algorithm of In_2H_x and In_3H_y (x = 0–4,6; y = 0–5) stoichiometries.

Stoichiometry	Population Size
In_2H_1	500
In_2H_2	1000
In_2H_3	1000
In_2H_4	1500
In_2H_6	1500
In_3	200
$In_3H_1^S$	750
$In_3H_1^T$	750
In_3H_2	1000
In_3H_3	1000
In_3H_4	1500
In_3H_5	3000

Final energies were calculated using QRO-CCSD(T)/cc-pVTZ level for the geometries obtained at U-TPSSh/def2-TZVPP level with Zero-Point energy corrections (ZPE). This calculation scheme is denoted as "QRO-CCSD(T)/cc-pVTZ(-PP) // U-TPSSh/def2-TZVPP".

Table S2. Spin-states' energy difference calculated at QRO-CCSD(T)/cc-pVTZ(-PP) // U-TPSSh/def2-TZVPP level of theory.

Stoichiometries	E^T - E^S, kcal/mol	Stoichiometries	E^Q - E^D, kcal/mol
In_2	-6.8	In_2H_1	30.3
In_2H_2	26.6	In_2H_3	52.5
In_2H_4	55.3	In_3	6.4
In_3H_1	1.4	In_3H_2	27.0
In_3H_3	23.2	In_3H_4	43.1
In_3H_5	36.8		

Table S3. In_2H_x and In_3H_y (x = 0–4,6; y = 0–5) global minimum geometries for singlet and doublet states found the lowest-lying isomers for triplet and quartet states (XYZ coordinates).

Stoichiometry	XYZ coordinates			
In_2^S	49	-0.644338000	1.214273000	0.881291000
	49	1.634438000	-0.263073000	-1.201191000
In_2^T	49	-0.517454000	1.132013000	0.765337000
	49	1.507554000	-0.180813000	-1.085237000

$In_2H_1^D$	49	-1.956105000	0.348048000	1.460731000
	49	-1.247821000	-0.607101000	-1.427238000
	1	-1.644175000	-1.412847000	0.430307000
$In_2H_1^Q$	49	0.000000000	0.000000000	-1.257087000
	49	0.000000000	0.000000000	1.370480000
	1	0.000000000	0.000000000	3.089901000
$In_2H_2^S$	49	0.094537000	-1.339387000	-2.245354000
	49	-2.254710000	-2.457598000	-0.140108000
	1	-1.613239000	-0.846706000	-1.229059000
	1	-0.546988000	-2.950309000	-1.156479000
$In_2H_2^T$	49	-1.311816000	-0.964966000	-1.240729000
	49	1.055717000	-0.022761000	-0.019842000
	1	-1.281758000	-0.971222000	-2.962632000
	1	-2.534943000	-1.435050000	-0.122496000
$In_2H_3^D$	49	1.472252000	-1.092080000	0.315474000
	49	-0.897219000	0.001864000	-0.752104000
	1	1.046641000	0.488827000	-0.843242000
	1	-0.221049000	-0.358585000	1.101607000
	1	0.037274000	-1.765726000	-0.913436000
$In_2H_3^Q$	49	-1.418372000	2.906147000	0.420144000
	49	-0.672003000	0.395574000	2.024225000
	1	-0.497513000	4.238049000	1.108992000
	1	-2.020709000	-0.722556000	1.865870000
	1	-0.319403000	1.291687000	0.321669000
$In_2H_4^S$	49	0.195760000	-0.950248000	1.370317000
	49	0.317625000	2.002374000	2.395903000
	1	0.393200000	-2.182850000	2.554988000
	1	-0.973741000	0.384486000	2.027872000
	1	-0.061536000	-1.166135000	-0.317560000
	1	1.476692000	0.414473000	1.650579000
$In_2H_4^T$	49	-0.999408000	2.545170000	-0.785135000

	49	-1.925037000	1.469252000	1.751409000
	1	-2.386625000	1.385320000	-0.178344000
	1	-3.084114000	2.485092000	2.488472000
	1	-0.492821000	2.652282000	1.051001000
	1	-1.174896000	-0.019216000	2.126895000
$In_2H_6^S$	49	-0.285229000	1.228282000	1.191348000
	49	-2.805268000	-0.290633000	1.442968000
	1	-2.085912000	1.224684000	0.464275000
	1	-1.004521000	-0.287084000	2.169960000
	1	0.754400000	0.612228000	-0.013800000
	1	-2.913798000	-1.598919000	0.352414000
	1	-3.844636000	0.325682000	2.648197000
	1	-0.176737000	2.536559000	2.281938000
In_3^D	49	1.532960000	1.593059000	-0.604616000
	49	-1.197470000	1.542509000	-1.557796000
	49	0.170810000	-0.932668000	-0.961288000
In_3^Q	49	0.190378000	0.599795000	2.540826000
	49	-2.051165000	0.377932000	-2.723139000
	49	-0.930313000	0.488773000	-0.091187000
$In_3H_1^S$	49	1.981057000	-1.169319000	0.867412000
	49	-0.074052000	0.001206000	-0.989637000
	49	-0.313259000	0.431519000	1.979083000
	1	-0.164845000	-1.310207000	0.717442000
$In_3H_1^T$	49	-0.908968000	-1.134072000	-1.836342000
	49	-3.192876000	-1.282413000	0.011548000
	49	0.166240000	0.015264000	0.647652000
	1	-1.726897000	0.289721000	-0.428058000
$In_3H_2^D$	49	2.067178000	0.512558000	1.073840000
	49	-0.099640000	2.504441000	0.702810000
	49	-0.865178000	0.304362000	-1.133466000

	1	1.035390000	1.340661000	-0.694274000
	1	-0.134749000	0.376880000	0.950491000
$In_3H_2^Q$	49	-0.443413000	1.180789000	1.204747000
	49	2.312139000	2.485607000	1.619583000
	49	1.502198000	0.353646000	-0.441427000
	1	-2.042950000	1.232606000	1.865480000
	1	2.208827000	-0.568147000	-1.725282000
$In_3H_3^S$	49	-1.408015000	1.068443000	-0.471232000
	49	0.533190000	-1.859373000	0.090137000
	49	1.774320000	0.776502000	-0.590546000
	1	0.084481000	-0.356345000	-1.385740000
	1	0.367652000	2.097817000	-0.811678000
	1	0.212272000	0.147454000	0.801259000
$In_3H_3^T$	49	-2.100191000	1.820666000	0.780997000
	49	-2.081756000	1.991848000	-2.423555000
	49	-0.349707000	-0.271996000	-0.946761000
	1	-0.457266000	1.122527000	-2.627334000
	1	-2.146639000	3.134741000	-0.965509000
	1	-2.579743000	0.575014000	-1.215038000
$In_3H_4^D$	49	-2.201962000	-0.737779000	-2.860178000
	49	-0.398762000	-2.360420000	-0.751927000
	49	1.020091000	-0.646209000	-3.070683000
	1	-1.933587000	-2.624108000	-1.686648000
	1	1.010868000	-2.543453000	-1.882216000
	1	-0.277344000	-2.994320000	0.830326000
	1	-0.473304000	-0.521512000	-1.120075000
$In_3H_4^Q$	49	-1.998663000	-1.548494000	-3.466103000
	49	-0.378127000	-1.896165000	-0.468959000
	49	0.731588000	-1.504318000	-3.690561000
	1	-1.981831000	-1.788372000	-1.577458000
	1	1.017605000	-1.747410000	-1.820593000

	1	-0.315011000	-3.523084000	0.075767000
	1	-0.329562000	-0.419957000	0.406506000
$In_3H_5^S$	49	-0.025583000	1.785443000	0.822966000
	49	-0.268775000	-1.298836000	-0.041061000
	49	2.362963000	0.023463000	-1.321271000
	1	1.782173000	1.045122000	0.318682000
	1	-0.089865000	0.237267000	-1.105625000
	1	-1.453339000	-2.483624000	-0.387584000
	1	-0.483504000	-0.292188000	1.452563000
	1	1.472731000	-1.734447000	-0.300870000
$In_3H_5^T$	49	2.864403000	1.328364000	-1.069743000
	49	1.984278000	0.729183000	1.912374000
	49	-0.209407000	0.277754000	-0.185289000
	1	1.094439000	0.720057000	-1.530882000
	1	2.973977000	3.079920000	-0.985766000
	1	0.763606000	-0.897974000	0.781190000
	1	0.046012000	1.584316000	1.050839000
	1	-1.683107000	-0.035718000	-0.985021000

Table S4. $NICS_{zz}$ values calculated at TPSSh/def2-TZVPP level of theory.

Molecule	$NICS_{zz}(0)$, ppm	$NICS_{zz}(1)$, ppm	$NICS_{zz}(2)$, ppm	$NICS_{zz}(3)$, ppm
**Benzene**	-15.199	-29.517	-17.315	-8.014
**In_3**	-2.146	-17.565	-17.139	-9.760
**In_3H_1**	+3.804	-12.155	-14.271	-8.235

AdNDP chemical bonding patterns and energy comparison of low-lying isomers

All bonding patterns are obtained at QRO-CCSD(T)/cc-pVTZ(-PP) // U-TPSSh/def2-TZVPP level of theory.

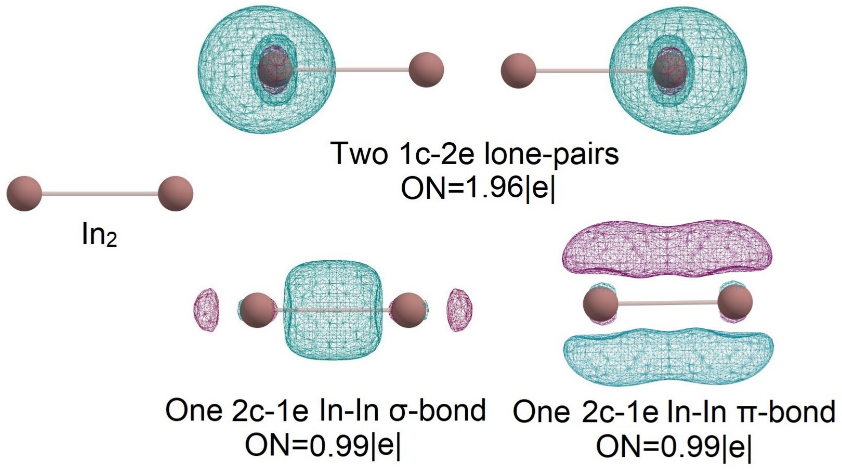

Figure S1. Chemical bonding pattern of GM (Global Minimum) structure of In_2.

For In_2H_1, we investigated two low-lying structures. In the case of both structures, we found lone pairs on all Indium atoms. In the GM structure, the H atom is connected to the In_2 fragment via one 3c-2e σ-bond, whereas in ISO1 H atom is connected via classical 2c-2e σ-bond with one Indium atom. In both isomers, Indium atoms are connected via 2c-1e In-In bonds.

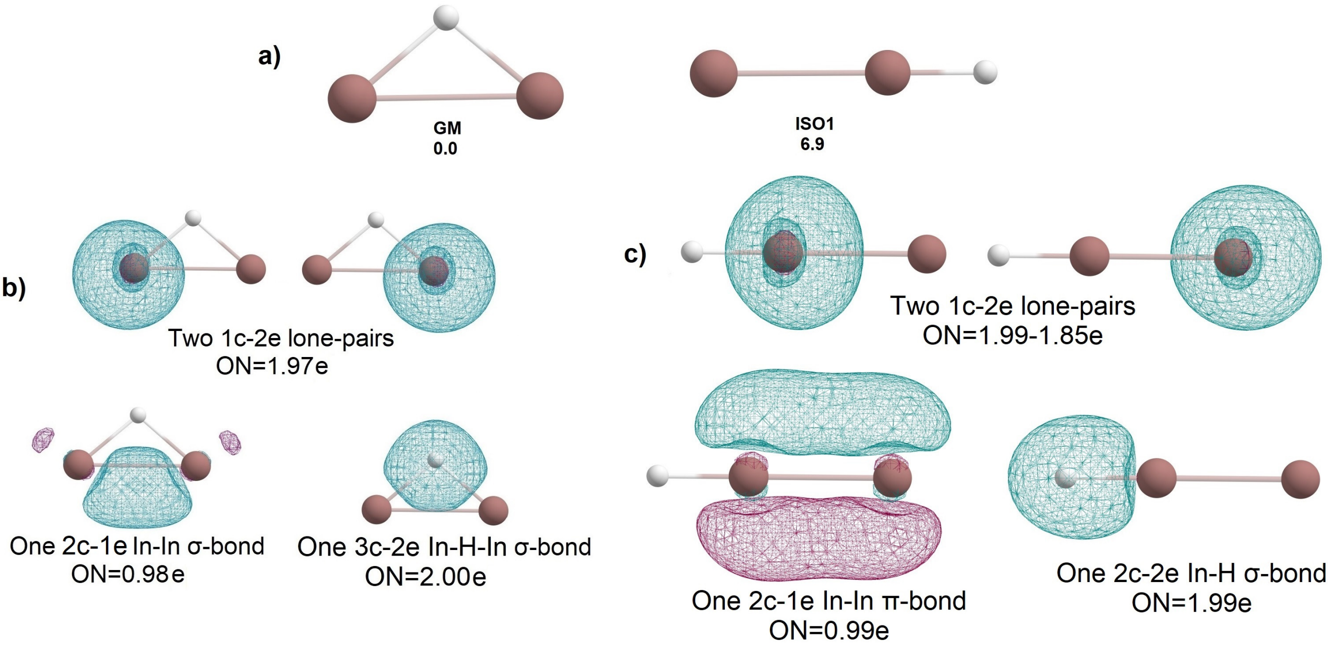

Figure S2. a) GM (Global Minimum) geometry and low-lying geometry of In_2H_1 with corresponding relative energies, b) chemical bonding pattern of GM structure of In_2H_1, c) chemical bonding pattern of ISO1 structure of In_2H_1.

For In_2H_2, we found several isomers. The ISO1 is much higher in energy than the GM structure. Both of them have lone pairs on each Indium atom. The bonding patterns of the two isomers are very similar; the GM has two 3c-2e σ-bond, whereas ISO1 has one 3c-2e σ-bond and one classical 2c-2e In-H σ-bond.

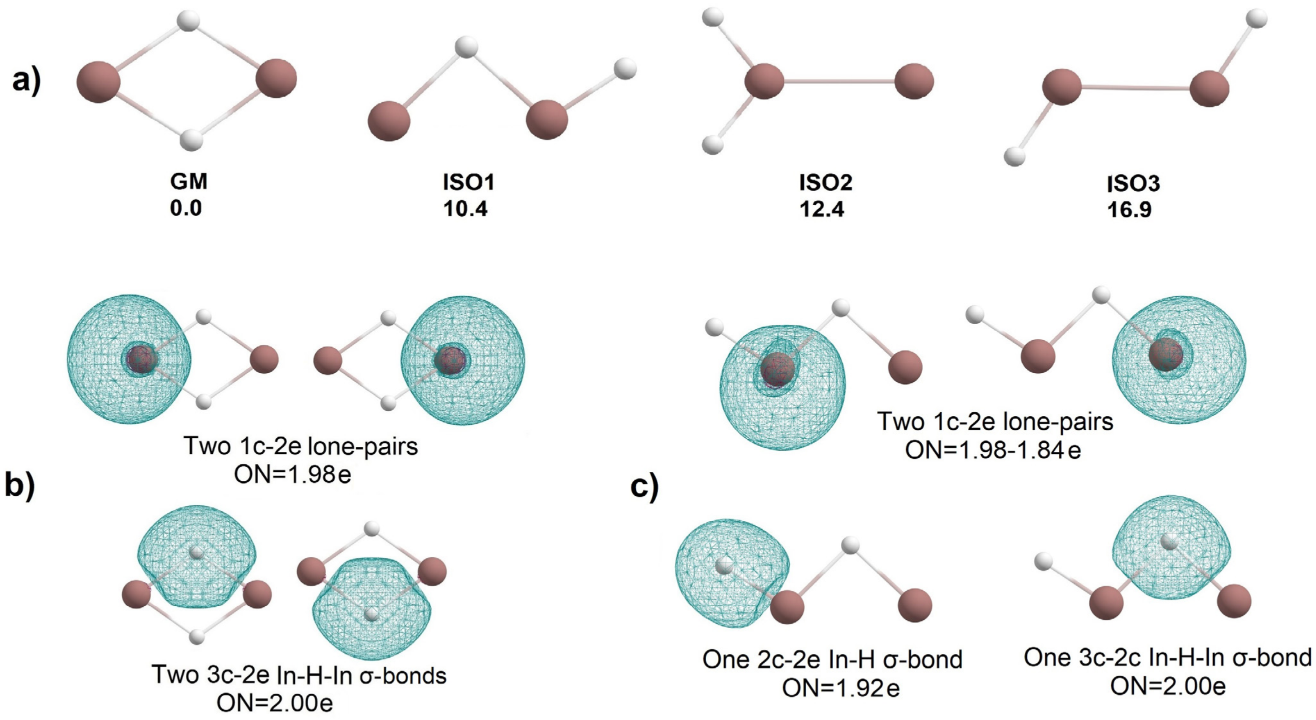

Figure S3. a) GM (Global Minimum) geometry and low-lying geometries of In_2H_2 with corresponding relative energies, b) chemical bonding pattern of GM structure of In_2H_2, c) chemical bonding pattern of ISO1 structure of In_2H_2.

In the case of In_2H_3 stoichiometry, the found low-lying isomers are relatively close in energy to the GM structure. We can see similar bonding patterns. One Indium atom has one 1c-2e lone pair, and another Indium atom has one 1c-1e unpaired electron in the case of both the GM and the ISO1 structures. The GM has three 3c-2e In-H-In σ-bond, the ISO1 has two 3c-2e In-H-In σ-bond and one classical 2c-2e In-H σ-bond.

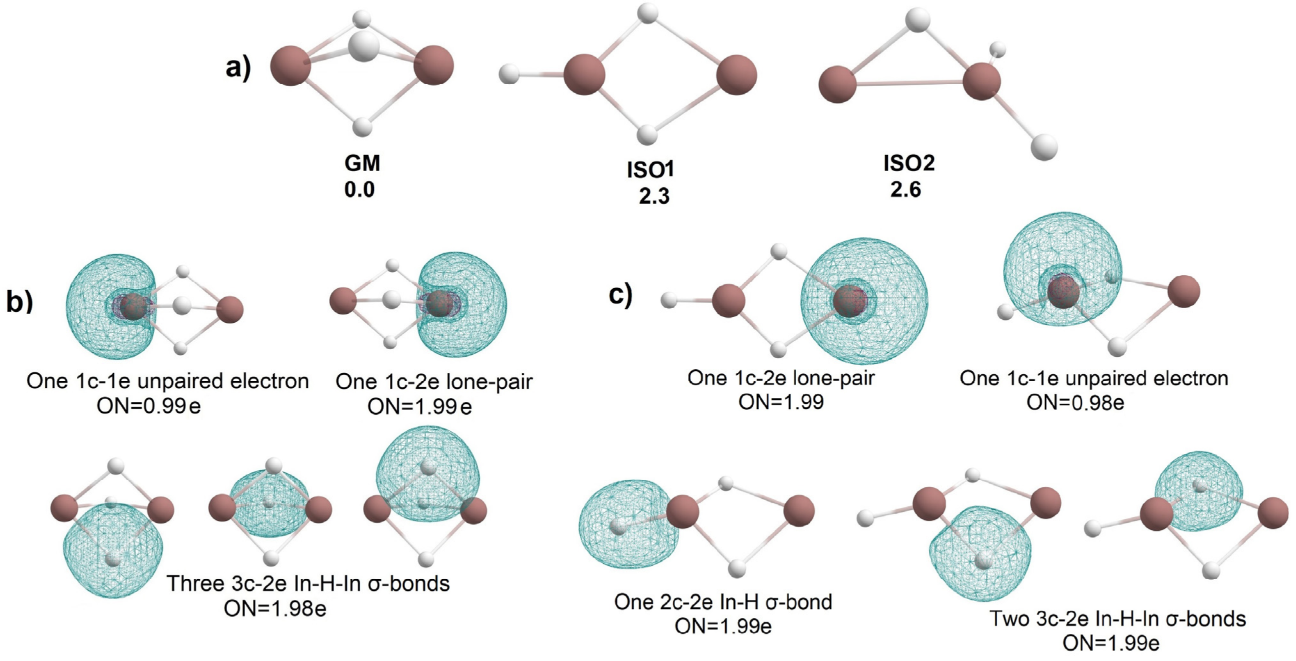

Figure S4. a) GM (Global Minimum) geometry and low-lying geometries of In_2H_3 with corresponding relative energies, b) chemical bonding pattern of GM structure of In_2H_3, c) chemical bonding pattern of ISO1 structure of In_2H_3.

For In_2H_4, we found two very energetically close isomers; all other isomers are much higher in energy. They have the same type of bonds. For both the GM and the ISO1, we found one 1c-2e lone pair. The GM has two In-H-In 3c-2e σ-bonds and two In-H 2c-2e σ-bonds, whereas the ISO1 has three 3c-2e In-H-In σ-bond and one 2c-2e In-H σ-bond.

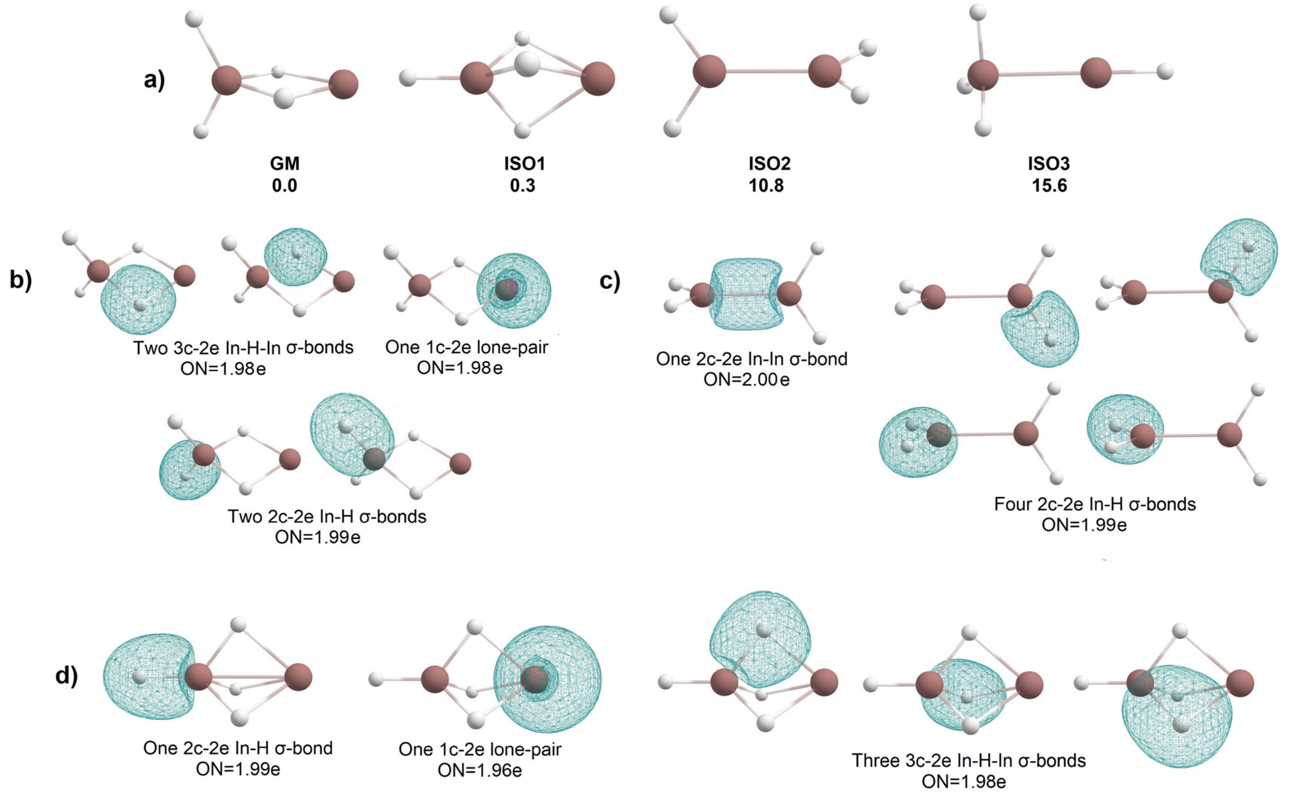

Figure S5. a) GM (Global Minimum) geometry and low-lying geometries of In_2H_4 with corresponding relative energies, b) chemical bonding pattern of GM structure of In_2H_4 (non-classical structure), c) chemical bonding pattern of classical structure of In_2H_4, d) chemical bonding pattern of ISO1 structure of In_2H_4.

For the In_3 cluster, we found two low-lying isomers. The GM is a triangular aromatic cluster with three 1c-2e lone pairs, one 3c-1e In-In-In σ-bond, and one 3c-2e In-In-In π-bond. However, the ISO1 has three 1c-2e lone pairs, one 3c-2e In-In-In σ-bond, and one 3c-1e In-In-In π-bond. Thus, the bond patterns of the isomers look similar.

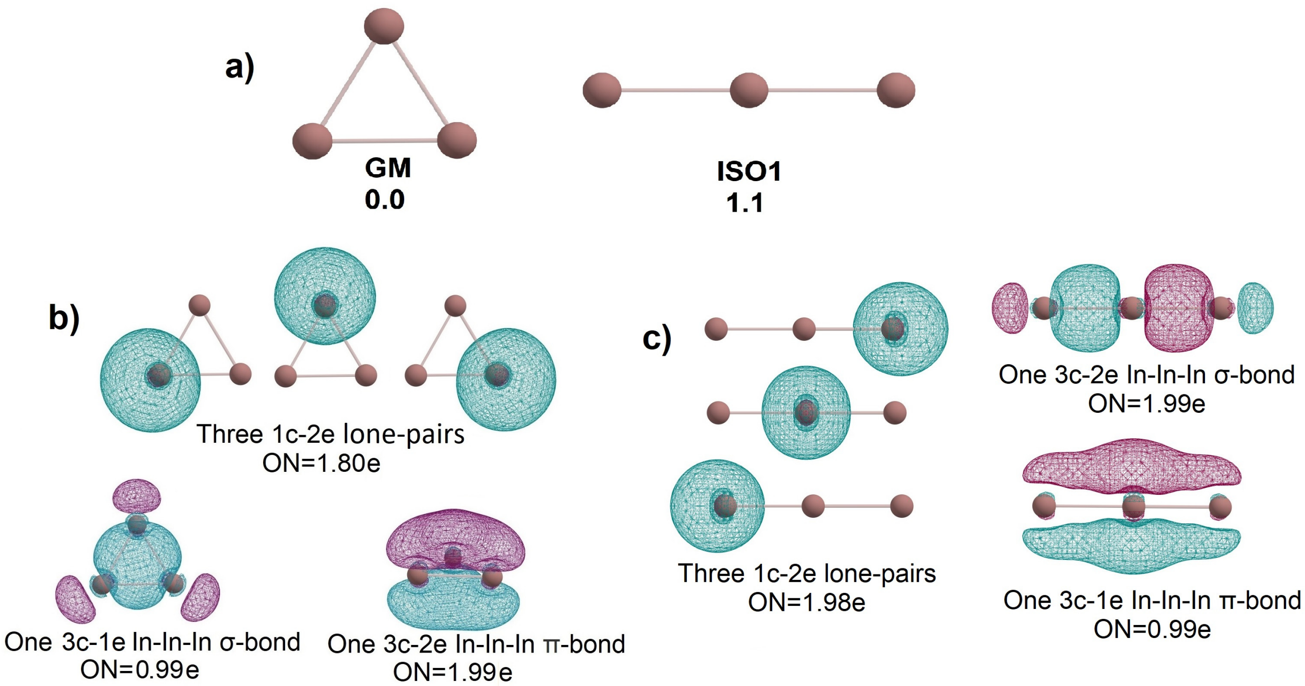

Figure S6. a) GM (Global Minimum) geometry and low-lying geometry of In_3 with corresponding relative energies, b) chemical bonding pattern of GM structure of In_3, c) chemical bonding pattern of ISO1 structure of In_3.

CK's search for the In_3H_1 stoichiometry revealed that GM and ISO1 are very close in energy, but other isomers' energies are much higher than GM's. The GM and the ISO1 are derivatives of the triangular In3 cluster; there are 1c-2e lone pairs on each Indium atom. The bounding determines the difference between the structure with the H atom. The GM has one 4c-2e In-In-In-H σ-bond, whereas the ISO1 has one 2c-2e In-H σ-bond.

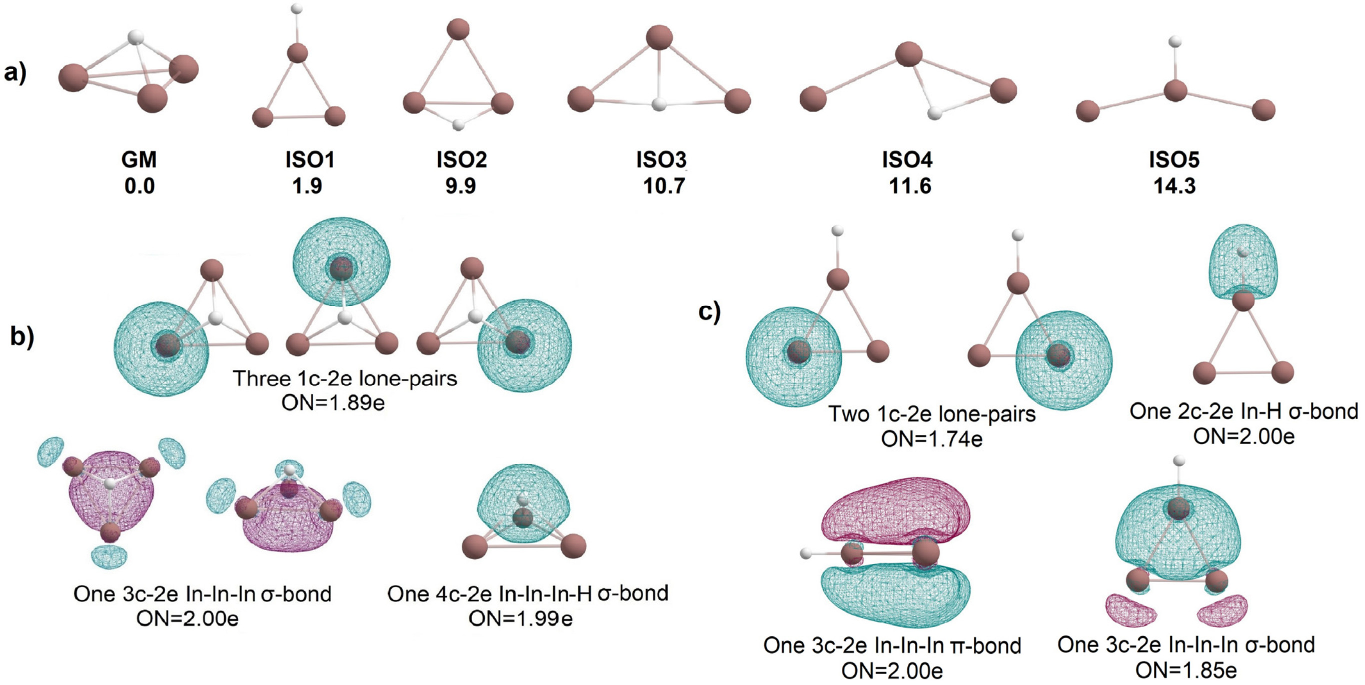

Figure S7. a) GM (Global Minimum) geometry and low-lying geometries of In_3H_1 with corresponding relative energies, b) chemical bonding pattern of GM structure of In_3H_1, c) chemical bonding pattern of ISO1 structure of In_3H_1.

For the In_3H_2 stoichiometry, we found several isomers. The GM structure has three 1c-2e lone pairs, two 4c-2e In-In-In-H σ-bonds, and one 3c-1e In-In-In σ-bond. The ISO1 also has three 1c-2e lone pairs. However, it has only one 4c-2e, one 3c-2e In-H-In σ-bond, and one 3c-1e In-In-In σ-bond. All H atoms are connected to the In_3 cluster via multi-center bonds.

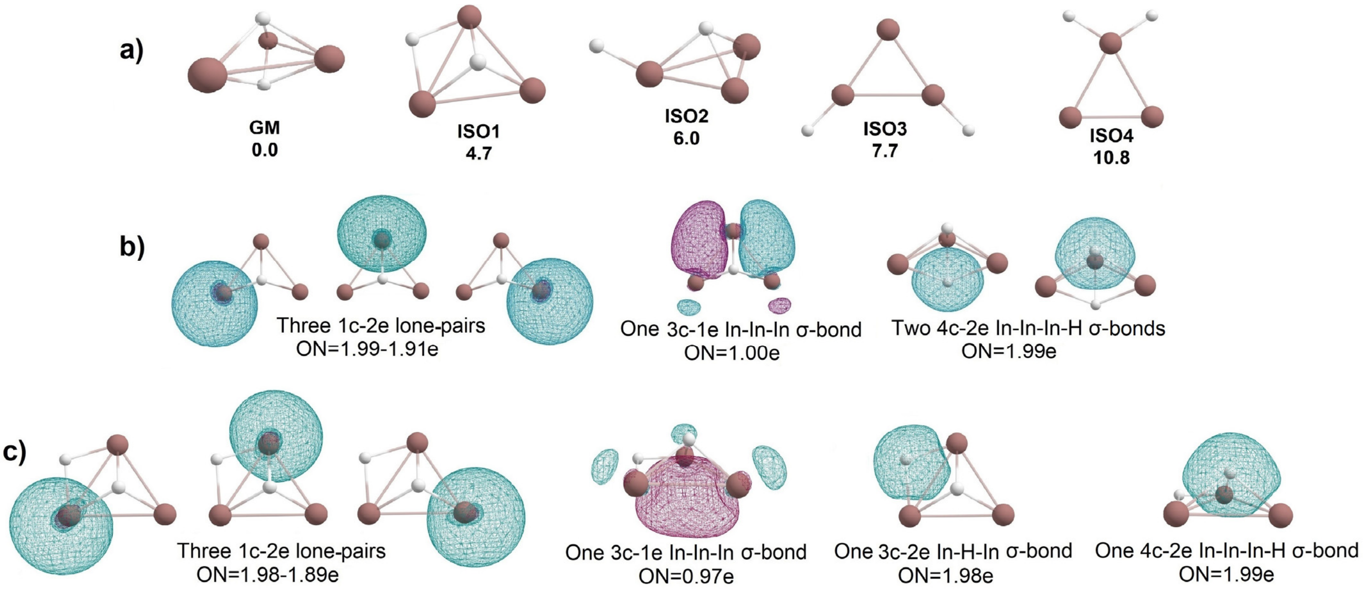

Figure S8. a) GM (Global Minimum) geometry and low-lying geometries of In_3H_2 with corresponding relative energies, b) chemical bonding pattern of GM structure of In_3H_2, c) chemical bonding pattern of ISO1 structure of In_3H_2.

There are a lot of close energy isomers for In_2H_3. The ISO1 and the GM structures have different structures because the GM is a derivative of the triangular In_3 cluster, whereas the ISO1 is a derivative

of the linear In_3 cluster. The GM has three 1c-2e lone pairs, two 4c-2e In-In-In-H σ-bonds, and one 3c-2e In-H-In σ-bond. The ISO1 has two 1c-2e lone pairs, three 3c-2e In-H-In σ-bonds, and one 2c-2e In-In σ-bond.

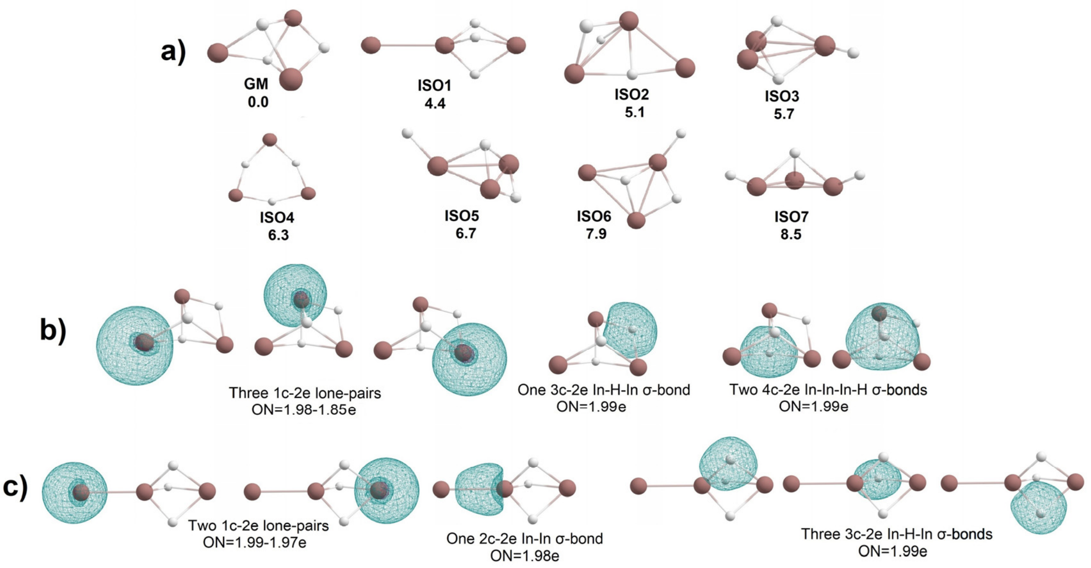

Figure S9. a) GM (Global Minimum) geometry and low-lying geometries of In_3H_3 with corresponding relative energies, b) chemical bonding pattern of GM structure of In_3H_3, c) chemical bonding pattern of ISO1 structure of In_3H_3.

We found plenty of isomers for In_3H_4 stoichiometries. Almost all of them are derivatives of the triangular In_3 cluster. The GM and the ISO1 are very close in energy. They have two 1c-2e lone pairs, one 2c-2e In-H σ-bond, and 2c-1e In-In σ-bond. However, the GM has two 3c-2e In-H-In σ-bonds and one 4c-2e In-In-In-H σ-bond, whereas the ISO1 has four 3c-2e In-H-In σ-bonds. So, the main difference between GM and ISO1 is the presence of 4c-2e In-In-In-H σ-bond in the GM structure.

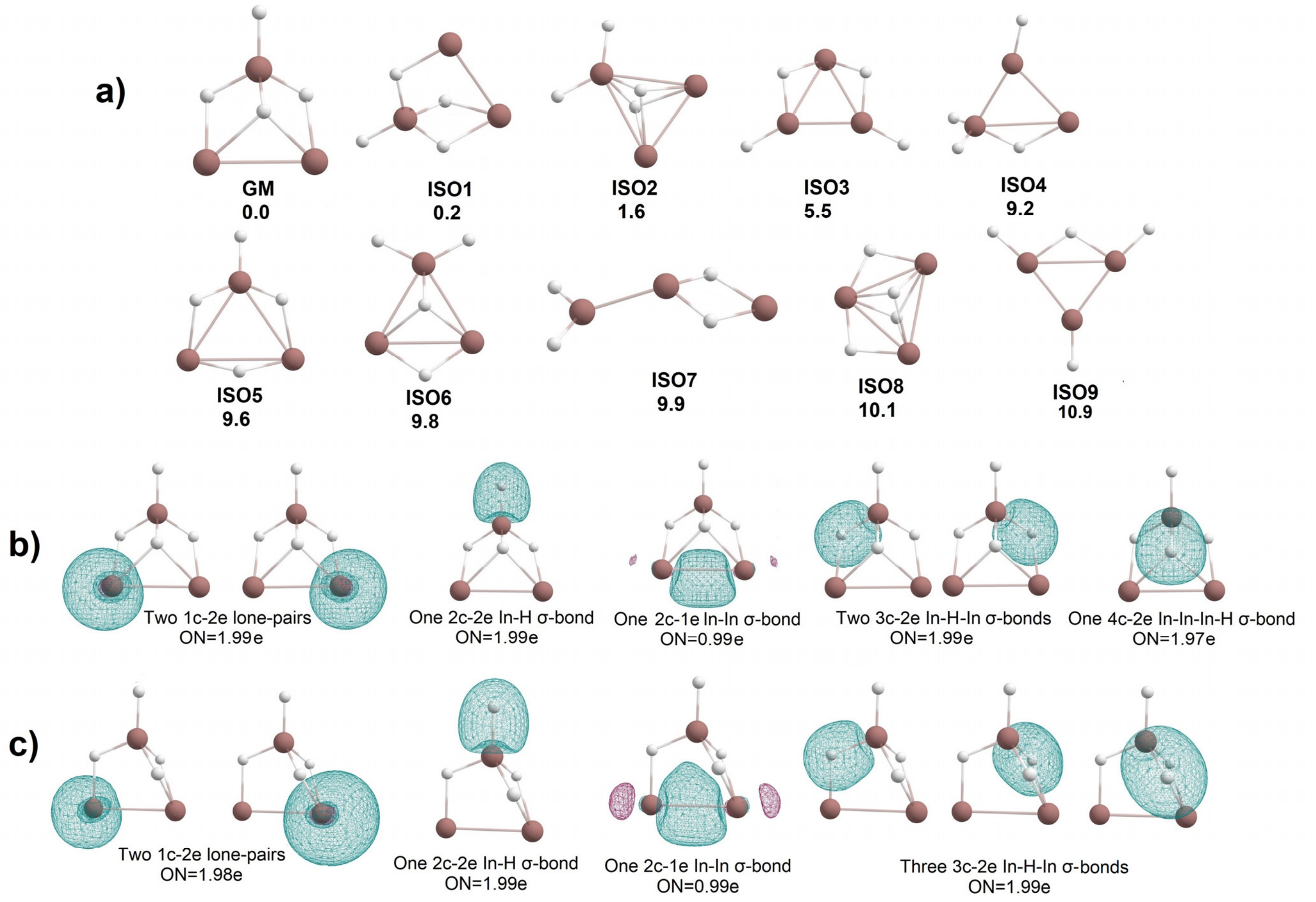

Figure S10. a) GM (Global Minimum) geometry and low-lying geometries of In_3H_4 with corresponding relative energies, b) chemical bonding pattern of GM structure of In_3H_4, c) chemical bonding pattern of ISO1 structure of In_3H_4.

As in the case of In_3H_4, we found many isomers for In_3H_5 stoichiometry. The ISO1 and the GM again are very close in energy; both of them have two 1c-2e lone pairs, one 2c-2e In-H σ-bond. The only difference is the 4c-2e In-In-In-H bond in the GM structure. Thus, the GM has three 3c-2e In-H-In σ-bonds, but the ISO1 has four 3c-2e In-H-In σ-bonds.

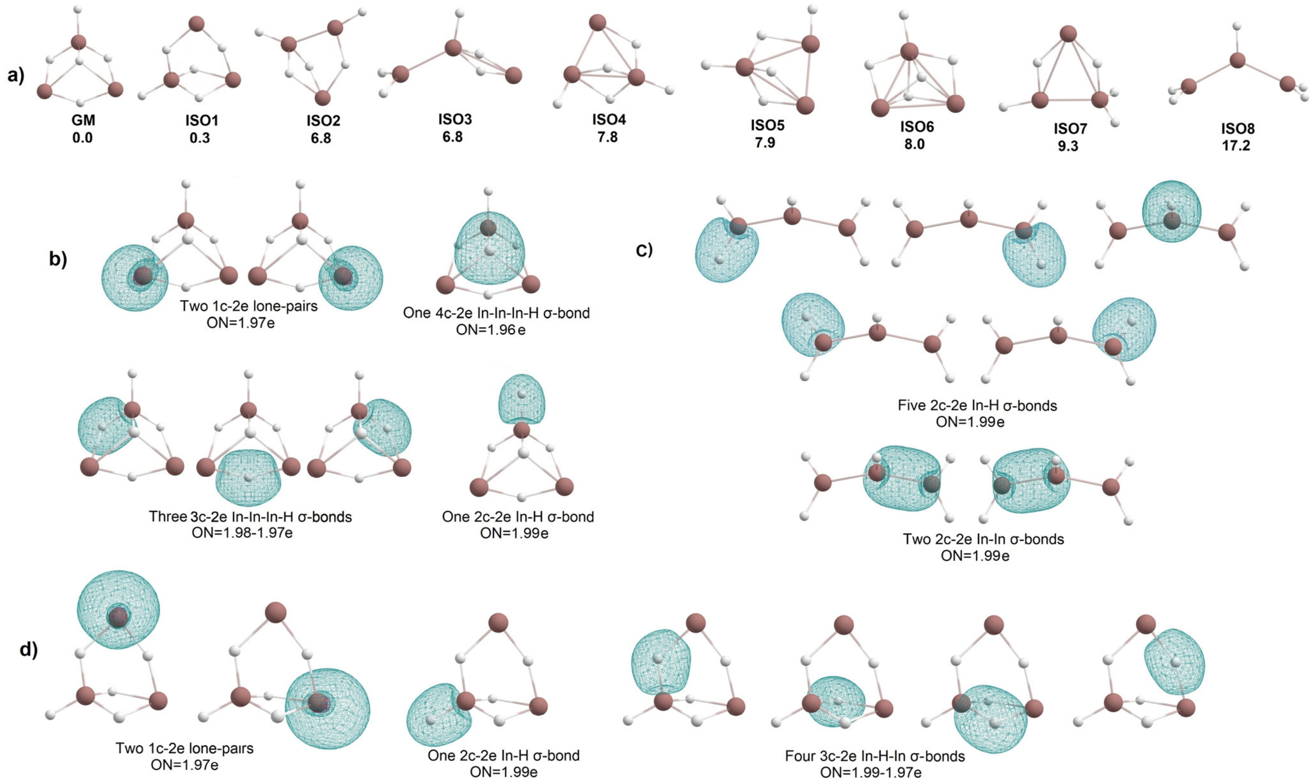

Figure S11. a) GM (Global Minimum) geometry and low-lying geometries of In_3H_5 with corresponding relative energies, b) chemical bonding pattern of GM structure of In_3H_5 (non-classical structure), c) chemical bonding pattern of classical structure of In_3H_5, c) chemical bonding pattern of ISO1 structure of In_3H_5.
